# Atrial Fibrillation and Cancer—Epidemiology, Mechanisms, and Management

**DOI:** 10.3390/jcm13247753

**Published:** 2024-12-19

**Authors:** Nathaniel E. Davis, Narut Prasitlumkum, Nicholas Y. Tan

**Affiliations:** 1 Department of Internal Medicine, Mayo Clinic, Rochester, MN 55905, USA; davis.nathaniel2@mayo.edu; 2 Department of Cardiovascular Medicine, Mayo Clinic, Rochester, MN 55905, USA

**Keywords:** atrial fibrillation, cancer, epidemiology, mechanisms, management

## Abstract

Atrial fibrillation (AF) and cancer are increasingly recognized as interrelated conditions, with cancer patients showing elevated incidences of AF, and there is evidence that AF may sometimes precede cancer diagnoses. This comprehensive review investigates the epidemiology, pathophysiology, and management challenges associated with AF in cancer patients. Epidemiologically, several cancers are more closely related to increased rates of AF, including lung, colorectal, gastrointestinal, and hematologic malignancies. Mechanistically, both AF and cancer share pathophysiological pathways centered on inflammation, oxidative stress, and common cardiovascular risk factors, such as hypertension, obesity, and diabetes. The inflammatory microenvironment in tumors, marked by increased cytokines and growth factors, promotes atrial remodeling and AF susceptibility. Elevated reactive oxygen species (ROS) levels, driven by the metabolic demands of cancer, further contribute to atrial fibrosis and structural changes. Moreover, many anticancer treatments exacerbate AF risk. Management of AF in cancer patients presents many unique challenges and requires a multidisciplinary approach. Rate and rhythm control strategies are complicated by potential drug–drug interactions and limited data surrounding early implementation of rhythm control strategies in cancer patients. Interventional approaches such as catheter ablation, though effective in maintaining sinus rhythm, carry significant perioperative risk in patients with malignancy. Stroke prevention with anticoagulants is essential but requires cautious administration to avoid heightened bleeding risks, particularly in patients undergoing chemotherapy. Further, the limited applicability of standard risk stratification tools like CHA2DS2-VASc in this population complicate decisions regarding anticoagulation. This review highlights the bidirectional relationship between AF and cancer, the difficulties in management, and the critical need for further research in this field.

## 1. Introduction

Atrial fibrillation (AF) and cancer, two prevalent and serious medical conditions, share intricate connections that have garnered increasing attention in recent years. While advances in cancer treatments have significantly improved patient outcomes and survival rates, the emergence of cardiovascular complications, including AF, has become a growing concern. In this comprehensive review, we explore the epidemiology between atrial fibrillation and cancer, including the higher rates of prevalence in malignancy, the possible bidirectional relationship that exists between the two conditions, and the impact atrial fibrillation has on morbidity and mortality. We also examine potential mechanisms driving the association such as the role of chronic inflammation, cardiac remodeling, and cancer directed therapy. Finally, we discuss the challenges and strategies in managing these comorbid conditions, providing information regarding rate vs. rhythm control, pharmacologic vs. interventional management, and considerations for anticoagulation in this patient cohort.

## 2. Epidemiology

*Risk of AF with cancer:* Numerous epidemiological studies have documented a bidirectional relationship between AF and cancer. Cancer patients, particularly those with advanced disease, have been shown to have an elevated risk of developing AF [1,2]. More specifically, lung, colorectal, gastrointestinal, genitourinary, and hematologic malignancies have shown the largest degree of association [3,4,5,6]. In one Danish cohort study, the prevalence of AF in the cancer cohort was 7.5% vs. 4.3% in the controls [1]. However, rates of AF vary dramatically across the literature, with incidence reported as high as 30% [7,8,9,10]. Conversely, emerging evidence suggests that AF may precede the diagnosis of cancer in some cases, raising intriguing questions about the temporal relationship between these two conditions. A retrospective cohort study by Conen et al. reported a higher incidence of subsequent cancer diagnoses among patients with AF compared to the general population, particularly within the first few months following AF diagnosis [11]. Further, a meta-analysis performed Yuan et al. recommended screening for occult malignancy in patients with new onset AF diagnosis given the significant relationship within the first three month period [12]. These results are controversial, and other studies examining the temporal relationship have contradicted these findings [5,13]. Further, patients with AF could be more likely to seek medical care due to their condition, potentially leading to increased opportunities for cancer detection. Other areas of possible confounding are differences in reporting based on race, lack of sex-stratified analysis, and lack of understanding regarding the role that social determinants of health play in care and outcomes in patients with AF [14,15]. While the underlying mechanisms driving this association remain incompletely understood, it is hypothesized that shared risk factors or pathophysiological pathways may contribute to the development of both AF and cancer.

*Impact on morbidity and mortality:* The coexistence of AF and cancer poses substantial challenges in terms of morbidity and mortality. AF significantly increases the risk of stroke; however, there is contradicting evidence regarding whether patients with AF in the setting of malignancy have an even greater risk of thromboembolic events. Several studies by Khamis et al., Hu et al., and Pastori et al. demonstrated increased risk of CVA [16,17,18]. However, in a study performed by Elbadawi et al., there was a lower risk of in hospital CVA in the cancer group when compared to the control group [19]. Other studies analyzing risk of stroke in this patient population showed similar findings [20,21]. Moreover, the use of anticoagulant therapy in these patients must be carefully balanced with the risk of bleeding, which can be exacerbated by cancer-related thrombocytopenia or coagulopathy. More specifically, one study performed by Pastori et al. showed cancer increases the risk of major bleeding (HR 1.27) and ICH (HR 1.07) in AF patients in a nationwide cohort study [16]. Further, the commonly used CHA_2_DS_2_VASC for risk stratification has been demonstrated to possibly provide a less accurate representation of overall stroke risk in patients with concomitant AF and malignancy [22]. AF may also complicate cancer management by necessitating modifications in treatment regimens, such as dose adjustments or the selection of specific chemotherapeutic agents to mitigate cardiac risks. Importantly, the presence of AF has been associated with worse overall prognosis and increased mortality rates in cancer patients, highlighting the clinical significance of this comorbidity [8,16,19,23].

### 2.1. Mechanisms

*Shared pathophysiology and risk factors:* Chronic inflammation and an increased burden of cardiovascular risk factors, such as hypertension, obesity, and diabetes, have been implicated in the pathogenesis of both AF and cancer [24,25,26,27]. However, there may be additional mechanisms specifically linking AF and cancer related to heightened inflammation often seen in the latter. The inflammatory milieu within the tumor microenvironment, characterized by the release of cytokines, chemokines, and growth factors, may promote atrial remodeling, development of atypical foci, and predispose to the development of AF [25,26,28,29,30]. In particular, there have been a number of malignancies associated with increased C-reactive proteins, interleukins, including IL-2, IL-6, and IL-8, macrophage migration inhibition factor, and tumor necrosis factor alpha (TNF-α), which are associated with systemic inflammation [31]. In general, increased inflammatory markers can lead to autonomic dysfunction, structural alterations of the heart, and electrical remodeling. One possible causal mechanism by which activation of the immune system mediates development of AF has been demonstrated through the NLRP3 inflammasome [32]. The NLRP3 inflammasome mediates caspase-1 activation and IL-1β release in immune cells. This interleukin has been seen at higher rates in cancer patients and plays a role in the development of AF, suggesting a possible concomitant mechanism.

Reactive oxygen species (ROS) represent another common avenue for the development of AF and cancer. ROS are a byproduct of increased cell metabolism and can promote atrial fibrosis and cardiac remodeling in the atrium [24,33,34]. Numerous studies have shown increases in ROS with malignancy [35,36,37]. Further, obesity and metabolic syndrome are independently associated with elevated levels of ROS, which may, in turn, contribute to the onset of both AF and malignancy [37,38,39,40]. Additionally, hypertension, obesity, and metabolic syndrome remain underdiagnosed in patients with cancer, and increased screening and treatment will be essential to minimize the role these chronic diseases play with regards to comorbid AF [41,42].

*Cancer therapies associated with AF risk:* Various pharmacologic cancer treatments have been implicated in the development of AF and are summarized in the Table 1 below [43,44,45,46].

**Anthracyclines** are a class of chemotherapy drugs commonly used to treat various types of cancer, including breast cancer, lymphoma, and leukemia. Numerous studies have demonstrated the relationship between anthracyclines and arrhythmia among other adverse cardiovascular events [47]. Additionally, development of AF while taking anthracyclines amplifies the risk of heart failure, compounding adverse event risk for patients [48]. While the exact mechanism is not fully understood, several hypotheses have been proposed, including cardiomyocyte damage secondary to oxidative stress, leading to cardiomyopathy and disruption of normal electrical conduction, ion channel dysfunction through direct interference, myocarditis with associated cardiac remodeling, and autonomic dysfunction, which can predispose individuals to arrythmias, including AF [49,50]. It is important to note that the cardiotoxic effects of anthracyclines are dose-dependent [51,52].

**Tyrosine kinase inhibitors**, particularly bruton tyrosine kinase inhibitors (TKIs) such as ibrutinib, are another class of anti-cancer therapy linked to increased risk of AF [33,53,54,55]. Ibrutinib, which is used to treat a variety of B-cell malignancies, has a well-documented association with AF. In one prospective study of CLL patients receiving ibrutinib by Matiello et al., 30% of patients had documented AF events [56]. Another review by Quartermaine et al. found rates of AF with ibrutinib for the treatment of CLL as high as 38% [57]. Increased AF risk (to varying degrees) have been seen with other TKIs as well [57,58,59]. Second- and third-generation Bruton TKI’s appear to have a lower rates of AF with lower rates of treatment interruption due to arrhythmias; however, long term data is sparse for these medications [60,61,62,63]. Additionally, there are other studies which have shown no difference in rates of adverse cardiac events or incidence of AF between different generations of Bruton TKIs [64].

The pathophysiology of ibrutinib or other TKIs leading to the development of AF may be related to off-target inhibition of C-terminal Src kinase, which leads to structural remodeling, myocardial fibrosis, and inflammation in the atrium [65]. These findings have been shown in animal models, as well as in clinical studies [65,66].

Other anticancer therapies have been associated with AF, but rates of incidence are not well established. **Alkylating agents** such as cisplatin, cyclophosphamide, and melphalan are one such class [67,68,69,70,71,72]. Cisplatin is thought to cause upregulation of TNF-α and NF-κB which leads to cardiotoxic effects [73]. Other proposed mechanisms for alkylating agents include alteration of intracellular calcium activity and aggravation of pre-existing pro-arrhythmic conditions [44]. Similarly to alkylating agents, **anti-metabolites**, including 5-fluorouracil (5-FU), gemcitabine, and capecitabine, are felt to be associated with AF, although incidence is poorly characterized. AF occurrence with 5-fluorouracil (5-FU) was seen in several studies and case reports [74,75]. However, rates appear to be low, with one retrospective study by Dyhl-Polk et al. demonstrating that 1.8% of patients developed symptomatic arrhythmias, including AF [76]. There are several proposed mechanisms through which 5-FU could lead to arrythmia, including endothelial dysfunction, vasospasm, mitochondrial damage and direct toxicity, and oxidative stress [77,78,79]. Gemcitabine was associated with AF in combination with vinorelbine, but not when used alone [72]. Whether this is due to a direct toxic effect or the known interaction with rapid delayed rectifier potassium channels is not understood [44,80]. Capecitabine, an oral prodrug of 5-FU, has documented cardiotoxicity, including arrhythmias, but the specific incidence of AF has not been well established [81]. One retrospective study of 452 patients with metastatic breast cancer had five incidences of AF [82].

**HER-2 antagonists** are well known for cardiotoxic effects, primarily heart failure and left ventricular dysfunction. These changes are mediated through several different mechanisms, including: disruption of HER2 signaling pathways leading to structural changes in the heart and; cardiac inflammation and fibrosis, disrupting normal cardiac architecture. Although the cardiotoxic impact of HER-2 antagonists is well documented, its direct relationship to the development of AF remains less clear. In a study by Wen-Chi et al., there was no increased risk of AF shown in breast cancer patients treated with HER-2 regimens [83]. Further, another study by Mauro et al. found no increase in AF incidence in breast cancer patients treated with HER-2 blockers [84].

CDK4/6 inhibitors are an emerging source of AF onset for patients being treated for breast cancer [85,86]. In a study performed by Fradley et al., adverse cardiovascular events, including AF, occurred in 24% of patients [85]. Further, no difference was seen in rates of AF between CDK4/6 inhibitors and anthracyclines. There are a number of mechanisms through which CDK 4/6 are thought to impact the cardiovascular system, including alteration of potassium and sodium channel activity, vascular inflammation, and downregulation of the PI3/AKT pathway [87,88,89]. However, more investigation is required to pinpoint the strongest drivers of AF development.

Rapidly accelerated fibrosarcoma B-type (BRAF) and mitogen-activated extracellular signal-regulated kinase (MEK) inhibitors are therapies utilized for management of melanoma. Although systematic capture and documentation of atrial arrhythmias have not been shown in clinical trials of either of these medications, there are a small number of studies that did show arrhythmia, most commonly AF [90,91]. The incidence of these events appears to be between 1 and 4% in patients treated with BRAF/MEK inhibitors [92]. In a meta-analysis comparing monotherapy with combination BRAF/MEK regimens, there was no difference in relative risk between the groups when looking specifically at AF, but elevated risks for cardiovascular adverse events in general were observed [93].

Medications that modulate the immune system, such as **immune checkpoint inhibitors**, lenalidomide, and CAR-, may lead to immune-related adverse events. For immune checkpoint inhibitors, the most serious clinical cardiovascular adverse event is myocarditis, which can manifest as arrhythmias, including AF [94,95]. However, the incidence of AF varies between studies, with rates ranging from 1.5% to 28.6% of patients [95,96]. The risk of cardiovascular adverse events, including myocarditis, may be increased with dual agent use; furthermore, there is variation in risk between different immune checkpoint inhibitors [97]. Hence, there is a strong need for further research to elucidate the specific risks and mechanisms for cardiovascular toxicity between the drugs in this class [98]. The most commonly associated immune checkpoint inhibitors appear to be anti-cytotoxic T-lymphocyte-associated protein 4 therapy such as ipilimumab [97,98,99]. While all immune checkpoint inhibitors enhance T cell activation and increase secretion of pro-inflammatory cytokines promoting arrhythmogenesis, anti-cytotoxic T-lymphocyte associated protein 4 therapy is thought to possibly carry a higher risk given potential interactions with CD4 + CD28-T cells [100]. This lineage of T-cell was highlighted in the pathogenesis of AF by Sulzgruber et al. CAR-T is primarily linked to AF through cytokine release syndrome and is highly associated with elevated levels of inflammatory markers such as IL-10 and TNF-alpha [101,102,103]. Finally, lenalidomide has been associated with higher incidences of AF when compared to other medications. The mechanism through which it causes AF is unknown but is believed to be linked to its more general cardiotoxic effects, including myocarditis, endothelial dysfunction, and hypercoagulability [104,105,106].

### 2.2. Surgery

Surgical management of malignancy has long been associated with significant risks of postoperative AF. Incidence varies with surgical modality, thoracic surgery representing the greatest risk [107,108,109]. Risk factors include age, preexisting cardiovascular conditions, and intraoperative factors, including transfusion and the extent and duration of the procedure performed [110,111,112]. Additionally, other identified post-procedural cardiac complications, such as cardiomyopathy, infection, anemia, pericarditis, and pleurisy, all represent potential triggers of post-operative AF. A multicenter randomized controlled trial investigated the use of colchicine to mitigate against AF triggered by perioperative inflammation; however, no decrease in perioperative AF was seen [113].

### 2.3. Radiation

Another well-established treatment modality that has been associated with AF is radiation. The first mechanism by which radiation can increase the risk of AF is through direct cardiotoxicity, particularly in patients receiving thoracic radiation. In one study, patients with esophageal cancer treated with radiation had a relative risk of 8.96 for developing AF, likely related to the close anatomic proximity of the esophagus to the left atrium [114]. Other studies have supported the link between direct cardiotoxicity and AF development [115,116]. Further, recent evidence showed that sparing of pulmonary dosing in non-small cell lung cancer decreases the incidence of AF suggesting a causal link between radiation and AF development [117].

### 2.4. Management

#### 2.4.1. Overview

**Figure 1:**
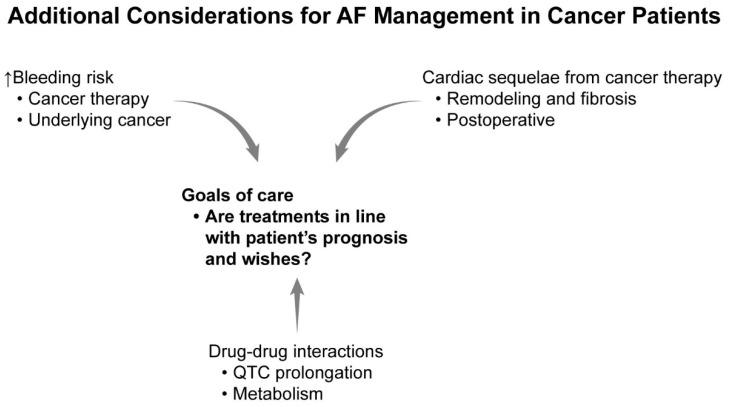
Considerations for atrial fibrillation management in cancer patients.

Treating AF in a cancer patient involves several considerations unique to the general population, where malignancy status/therapies and overall prognosis must be taken into account. These are summarized in Figure 1.

#### 2.4.2. Rate Versus Rhythm Control

While both rate and rhythm control strategies may be utilized, the selection of the optimal strategy should be guided by the goals of therapy, symptom burden, and the presence of underlying structural heart disease.

#### 2.4.3. Rate Control Medical Management

Medical therapies, such as beta-blockers, calcium channel blockers, and antiarrhythmic drugs, may be employed for rate control, while catheter ablation or pharmacological cardioversion may be considered for rhythm control in select patients. Per the 2022 European Society of Cardiology, beta blockers are the preferred therapy for rate control given the negative inotropic effect and potential drug–drug interactions of calcium channel blockers [118,119,120].

#### 2.4.4. Rate Control via Ablation

AV nodal ablation can be utilized for rate control in patients with AF who have failed rate control with medical therapy. A study performed by Peng et al. showed that AV node ablation resulted in no significant difference in recurrence, thromboembolism, bleeding, or mortality when conducted in cancer versus non-cancer patients [121]. Several other studies, including one performed on patients with neuroendocrine tumors and another on postpneumonectomy patients, demonstrated the feasibility and safety of AF ablation as a treatment modality [122,123]. However, there are several differences between cancer and non-cancer populations when considering ablation, particularly in peri-procedural outcomes and clinically relevant bleeding [124]. In a recent meta-analysis performed by Costa et al., an increased risk of bleeding after catheter ablation for AF in patients with cancer was seen, but there was no significant difference in efficacy for the maintenance of sinus rhythm between patient populations, emphasizing that while ablative modalities are effective, they carry increased risk for cancer patients [125].

#### 2.4.5. Rhythm Control Medical Management

Recent data have shown that institution of early rhythm control (i.e., within 1 year of diagnosis) is associated with decreased adverse cardiovascular outcomes, especially in the setting of heart failure and left ventricular systolic dysfunction. As such, contemporary AF guidelines have advocated for pursuing rhythm control strategies sooner, although it is not clear if such recommendations apply to cancer patients as well. Antiarrhythmic drugs are associated with QT-interval prolongation and frequently have interactions with cancer therapies [126,127]. Another complicating factor of pharmacologic rhythm control, particularly amiodarone, is significant non-cardiac toxicities [128,129]. Propafenone and sotalol are other options but require extensive interdisciplinary collaboration and caution due to potential of proarrhythmia, potential interaction with anticancer therapies, and required dose adjustments for renal function [130]. Antiarrhythmic medications and their potential interactions with cancer therapy are summarized in the table below. Amiodarone is a potent inhibitor of CYP3A4 and CYP2C9. Cyclophosphamide, doxorubicin, tamoxifen, and paclitaxel are metabolized through CYP3 enzymes, which are inhibited by amiodarone; hence, drug concentrations can be increased, leading to potentiation of toxicity [131,132,133]. Dofetilide, a class III antiarrhythmic, has the potential to prolong the QT interval. Several chemotherapy regimens are also associated with this affect, including anthracyclines, tyrosine kinase inhibitors, and 5-FU [134]. Further, certain chemotherapeutic agents. such as cisplatin, platinum actinide agents, and tyrosine kinase inhibitors, can inhibit MATE1, a crucial transporter for renal clearance of dofetilide [135,136,137]. Dronedarone interacts with various chemotherapies through the inhibition of CYP2D6, P-glycoprotein, and metabolism by CYP3A4 [138,139]. Disopyramide relies on CYP3A4 for metabolism and inhibition via tyrosine kinase inhibitors, and some chemotherapeutic agents can increase the risk of arrythmia [140,141]. Similarly, flecainide and propafenone concentrations and risk of toxicity is impacted by therapies that inhibit CYP3A4 and CYP2D6 pathways [134,142]. Finally, sotalol is renally cleared and thus avoids potential CYP enzyme interactions [143]. However, compounding QT prolongation when employing sotalol and certain chemotherapy regimens is a potential source of arrhythmia [134,144]. Table 2 summarizes the antiarrhythmic agents and their possible interactions.

#### 2.4.6. Rhythm Control via Ablation

Catheter ablation has emerged as the first-line option for AF rhythm control, especially in the context of heart failure [30]. Similar efficacy for catheter ablation in the maintenance of sinus rhythm for patients with and without cancer has been shown [145,146]. Limited data investigating the efficacy/safety profile in cancer patients have been obtained, although a retrospective study utilizing the National Readmissions Database highlighted increased odds of periprocedural complications and bleeding compared to non-cancer patients [147]. Increased risk of periprocedural complications has been highlighted in other studies as well [148,149]. Additionally, procedural radiation use may prompt concerns about triggering or exacerbating malignancies in patients [150]. However, continued advancements in technology, such as three-dimensional electroanatomic mapping, intracardiac ultrasound, and pulsed field ablation, may help to ameliorate the safety concerns of performing catheter ablation procedures in cancer patients, thereby “keeping on the table” invasive but effective therapies for them [151,152].

#### 2.4.7. Device Implantation

In conjunction with the increased periprocedural risks noted in ablation for cancer patients, device implantation has been demonstrated to carry increased risk of major adverse cardiovascular evens, all-cause mortality, major bleeding, and thoracic complications [153]. The risk of complication varies by malignancy, with the highest rates reported in hematologic and lung malignancies [153]. Modified risk profiles for patients with malignancy is also important to consider given the increasing use of leadless pacemakers and varying rates of complication when compared to traditional transvenous pacemakers. Traditionally, leadless pacemakers are more associated with vascular complications and venous thromboembolism, while transvenous pacemakers have greater rates of lead and infection related complications [154,155,156,157]. When looking at long term outcomes, leadless pacemakers have demonstrated overall lower rates of chronic complication and reintervention, with one three-year follow-up study showcasing a 32% lower rate of chronic complications and a 41% lower rate of reintervention when compared to transvenous pacemakers [158]. In high-risk groups, such as those with malignancy, leadless pacemakers again had lower rates of complication [159]. Finally, an integral consideration for all cancer patients considering pacemaker implantation is the future necessity of radiotherapy. Several studies have shown that radiation can be safely delivered to patients with implanted devices but emphasize the careful planning and multidisciplinary approach to management in these patients [160,161,162,163].

#### 2.4.8. Stroke Prevention

The presence of cancer-related hypercoagulability, the concurrent use of antineoplastic agents, and the potential drug interactions with anticoagulant therapy must be considered when determining the appropriateness of stroke prevention strategies. Assessing the risk of stroke in cancer patients with AF presents unique challenges, as traditional risk stratification tools, such as the CHA2DS2-VASc score, may not fully capture the thromboembolic risk profile in this population [21,164,165]. In one retrospective cohort study directly comparing patients with CHA2DS2-VASc < 2 in patients with and without cancer, 12-month cumulative incidences of arterial thromboembolism were 2.13% (95% CI: 1.47–2.99) in 1411 AF patients with cancer and 0.8% (95% CI: 0.56–1.10) in 4233 AF patients without cancer (HR: 2.70; 95% CI: 1.65–4.41) [166]. However, a meta-analysis comparing risk of arterial thromboembolism, bleeding, and mortality in 3,149,547 patients with AF and comorbid cancer vs. AF alone showed no increased risk of arterial thromboembolism but did show increased risk of bleeding and all-cause mortality [167]. The associations varied between malignancy, and further research is likely needed to elucidate cancer-specific risk for arterial thromboembolism and bleed.

Even with the unanswered questions regarding risk of venous thromboembolism, bleeding, and arterial thromboembolism, the foundation of stroke prevention remains anticoagulation with warfarin or direct oral anticoagulants (DOACs). However, there are a number of considerations that must be made prior to initiation of anticoagulation. There is a plethora of research showcasing similar efficacy with potentially improved safety profiles of DOACs when compared to warfarin [168,169,170,171,172,173]. There is discrepancy between recently published work regarding differences in adverse outcomes, including bleeding and hemorrhagic stroke, when comparing warfarin and DOACs. Some studies have shown apixaban carries the lowest risk of severe bleeding when compared to warfarin, while others found no statistically significant difference in rates of severe bleeding or other adverse events [169,170]. However, a recent meta-analysis conducted by Parrini et al. revealed no difference between various DOACs with regards to bleeding and showed lower rates adverse events but similar efficacy when compared to warfarin [168].

When employing DOACs or vitamin K antagonists, it is also important to recognize the potential drug–drug interactions, particularly with anti-neoplastic agents. The American Society of Hematology specifically highlights the potential for interaction with medications that affect P-gp and cytochrome P450, specifically CYP3A4, pathways [174]. Examples of medications that can interact with DOACs through P-gp pathways include tyrosine kinase inhibitors such as erlotinib or nilotinib, although the degree of interaction varies between medications within this class [175,176]. Other medications such as tamoxifen, irinotecan, and cyclosporine appear to be associated with higher risks of bleed when given with DOACs, possibly due to their role in the cytochrome and P-gp pathways [176]. Although the American Society of Hematology recommends utilizing vitamin K antagonists in the setting of medications that significantly impact P-gp and CYP3A4, these medications also have potential for interaction with anti-neoplastics such as 5-FU and capecitabine, among others [174,177,178]

While oral anticoagulants, such as warfarin or DOACs, remain the cornerstone of stroke prevention in AF, their use must be carefully weighed against the risk of bleeding, particularly in patients with active malignancy or undergoing invasive procedures. Left atrial appendage occlusion (LAAO) may offer an alternative stroke prevention strategy in selected patients who are deemed unsuitable for long-term anticoagulation. Kumar et al. found that LAOO device implantation resulted in similar long-term outcomes with regards to mortality, stroke, and major bleeding between cancer and non-cancer patients [179]. While Zhang et al. reported higher rates of certain acute procedural complications including pericardial drainage and bleeding, readmission rates were comparable between cancer and non-cancer patients [180]. Another study conducted by Shabtaie et al. demonstrated a reduction in overall stroke risk, without an increased risk of bleeding compared to non-cancer patients [181]. Overall, current evidence supports that LAOO provides an appropriate option for management of stroke risk in cancer patients with underlying AF, particularly in those with contraindications to anticoagulation.

#### 2.4.9. Suggested Management Algorithm

**Figure 2:**
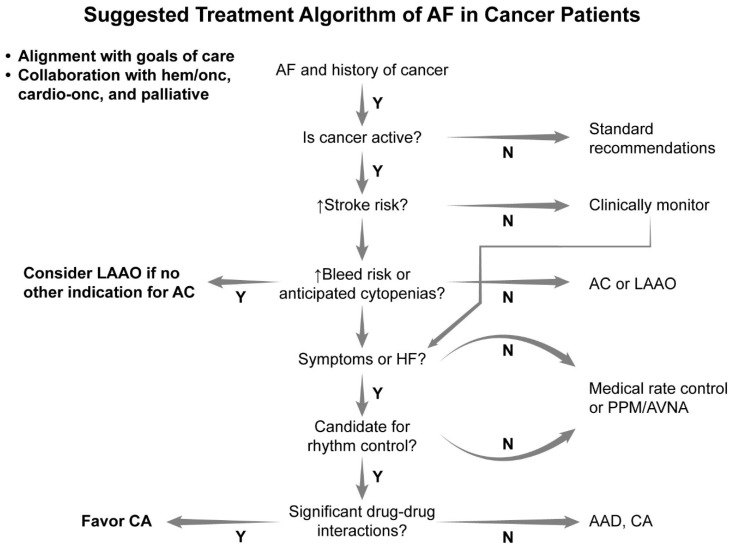
Suggested treatment algorithm of atrial fibrillation in cancer patients.

Figure 2 shows a schematic for treating AF that incorporates the elements discussed above. Where possible, collaboration with other specialized teams, including the primary hematologist/oncologist, is encouraged. Additionally, goals of care, in lieu of the overall prognosis, should always be kept in mind during the decision-making process.

#### 2.4.10. Primary and Secondary Prevention

The prevention of AF in patients should primarily be focused on minimizing the risk factors highlighted previously. However, there are several pharmacologic approaches which have been investigated to prevent occurrence. The first is amiodarone, particularly in the context of lung resection and esophagectomy. Several studies, including randomized controlled trials, have shown reduced incidence of AF when implementing postoperative prophylaxis with amiodarone [182,183,184,185]. The European Heart Association and ACC/AHA guidelines agree that amiodarone can be used in this context, but it requires careful monitoring for negative side effects [186,187]. Another therapeutic area of interest is tocilizumab in the context of CAR-T for cytokine release syndrome (CRS). While there appears to be a relationship between duration between CRS onset, administration of tocilizumab, and cardiovascular events, there is no evidence that it specifically prevents atrial fibrillation [188].

## 3. Conclusions

**Figure 3:**
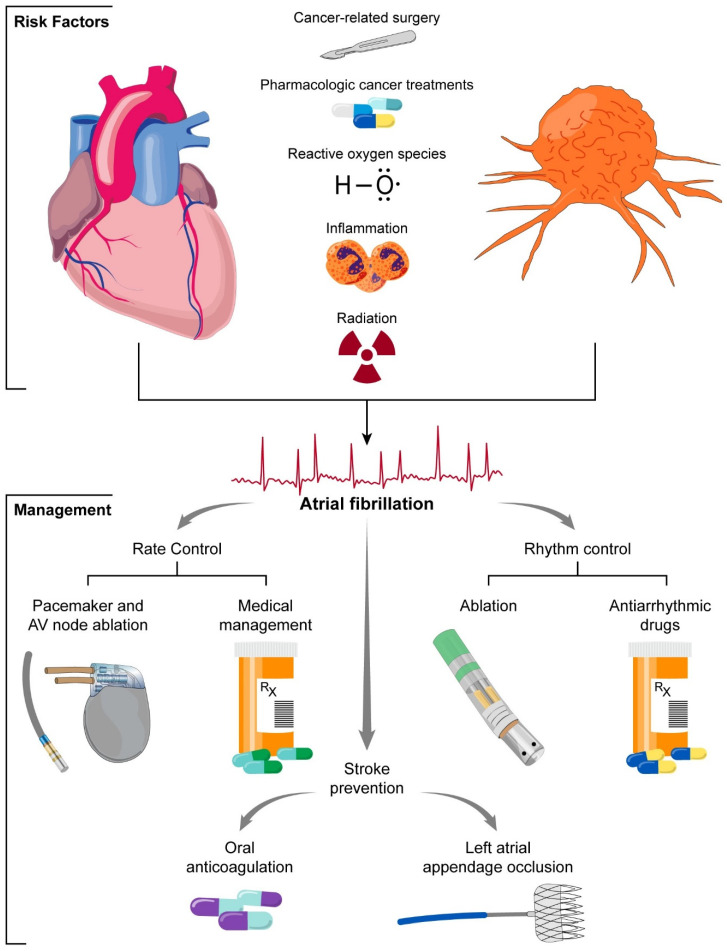
Central illustration.

In conclusion, the bidirectional relationship between AF and cancer represents a complex interplay between two prevalent and clinically significant conditions. While epidemiological studies have provided insights into the association between AF and cancer, further research is warranted to elucidate the underlying mechanisms driving their interaction and to develop targeted management strategies aimed at optimizing outcomes in this vulnerable population. Beyond conventional therapies, future developments in mobile and digital health could allow for better identification and expedited care in this vulnerable patient population. A multidisciplinary approach, involving collaboration between oncologists, cardiologists, and other healthcare providers, is essential for the comprehensive management of patients with concomitant AF and cancer (Figure 3).

## Figures and Tables

**Table 1:** Cancer drug classes and proposed mechanisms contributing to atrial fibrillation development.

Drug Class	Examples	Proposed Mechanisms
Anthracyclines	Doxorubicin, epirubicin, daunorubicin, idarubicin	Oxidative stress-induced cardiomyocyte damage, ion channel dysfunction, myocarditis and cardiac remodeling, autonomic dysfunction
Tyrosine Kinase Inhibitors	Ibrutinib, acalabrutinib, sorafenib, regorafenib	Off-target inhibition of C-terminal Src kinase, structural remodeling and myocardial fibrosis in the atrium, inflammation
Alkylating Agents	Cisplatin, cyclophosphamide, melphalan	TNF-α and NF-κB upregulation, alteration of intracellular calcium activity, aggravation of pre-existing pro-arrhythmic conditions
Anti-metabolites	5-fluorouracil (5-FU), gemcitabine, capecitabine, decitabine	Endothelial dysfunction and vasospasm, oxidative stress, direct myocardial toxicity, electrophysiologic change
HER-2 Antagonists	Trastuzumab	Disruption of HER2 signaling pathways, structural changes in the heart, cardiac inflammation, and fibrosis
CDK4/6 Inhibitors	Palbociclib, ribociclib	Alteration of potassium and sodium channel activity, vascular inflammation, downregulation of PI3/AKT pathway
BRAF/MEK Inhibitors	Vemurafenib, dabrafenib	Structural and electrical remodeling in the heart
Immune Checkpoint Inhibitors	Pembrolizumab, nivolumab	Myocarditis, cardiac inflammation leading to arrhythmias, variable incidence based on specific drug and combination therapy
CAR-T Cell Therapies	Axicabtagene ciloleucel, tisagenlecleucel	Cytokine release syndrome, elevated inflammatory markers (e.g., IL-10, TNF-alpha)
Lenalidomide		General cardiotoxic effects (exact mechanism for AF is unknown)

**Table 2:** Cancer drug classes associated mechanisms of interaction.

Drug Class	Potential Cancer Medications Impacted	Mechanism of Interaction
Amiodarone	Cyclophosphamide, doxorubicin, paclitaxel, tamoxifen, imatinib, erlotinib	Inhibition of CYP3A4, CYP2C9, CYP2D6, P-glycoprotein inhibition
Dofetilide	Anthracyclines, tyrosine kinase inhibitors, 5-FU, cisplatin, platinum actinide agents, Ivosidenib, ribociclib	QT prolongation, inhibition of MATE1
Dronedarone	Tyrosine kinase inhibitors (erlotinib, imatinib), vincristine, paclitaxel, tamoxifen	Metabolism by CYP3A4, moderate inhibition of CYP2D6 and P-glycoprotein
Disopyramide	Anthracyclines, tyrosine kinase inhibitors, cyclophosphamide, tamoxifen, irinotecan, docetaxel, Ivosidenib, ribociclib	QT prolongation, CYP3A4 inhibition
Flecainide	Anthracyclines, trastuzumab, tyrosine kinase inhibitors, tamoxifen, cobimetinib, ivosidenib, ribociclib	QT prolongation, CYP2D6 inhibition, CYP3A4 inhibition
Propafenone	Anthracyclines, trastuzumab, tyrosine kinase inhibitors, tamoxifen, cobimetinib	CYP3A4 inhibition, CYP2D6 inhibition
Sotalol	Anthracyclines, tyrosine kinase inhibitors, ivosidenib, ribociclib	QT prolongation

## Data Availability

Not applicable.
